# Spoof detection with dynamic learnable sparse attention and tri-modal fusion in resource-constrained audio systems

**DOI:** 10.1371/journal.pone.0335240

**Published:** 2025-12-31

**Authors:** Xinwei Wang, Zhicheng Tan, Guo Li

**Affiliations:** 1 Department of Forensic Science, Fu Jian Police College, Fuzhou, Fujian, China; 2 School of Aeronautics and Astronautics, Guilin University of Aerospace Technology, Guilin, China; Institute of Theoretical and Applied Informatics Polish Academy of Sciences: Instytut Informatyki Teoretycznej i Stosowanej Polskiej Akademii Nauk, UKRAINE

## Abstract

Audio sensors, essential for automatic speaker verification (ASV) systems, face growing threats from spoofed audio generated by advanced speech synthesis techniques. Traditional spoof detection methods, such as those based on computationally intensive Multi-Head Attention (MHA), suffer from quadratic complexity (*O*(*T*^2^)) and high memory demands, making them impractical for deployment on resource-constrained audio sensors. To address these limitations, we propose a novel Dynamic Learnable Sparse Attention (DLSA) framework that integrates Mel-Frequency Cepstral Coefficients (MFCC), Constant-Q Transform (CQT), and raw waveform modalities for spoof detection. The DLSA module introduces a learnable attention mechanism that dynamically selects key spectral and temporal features from MFCC and CQT for cross-modal fusion. A ResNet backbone is used to extract features from the raw waveform. We also introduce a hybrid loss function combining cross-entropy loss (${ℒ}_{CE}$) and center loss (${ℒ}_{center}$), optimizing intra-class compactness and inter-class separability. Compared to MHA-based methods, our approach reduces computational costs by 80%. Experimental results on the ASVspoof 2019 Logical Access (LA) dataset demonstrate a significant performance boost, achieving an Equal Error Rate (EER) of 0.68% and a minimum tandem Detection Cost Function (t-DCF) of 0.0173, outperforming existing methods by 33.6% in EER reduction. This approach provides an efficient and robust solution for spoof detection in resource-constrained ASV systems.

## Introduction

The advent of sophisticated speech synthesis and voice manipulation technologies has revolutionized human-computer interaction, enabling applications ranging from virtual assistants to entertainment [1,2]. However, this technological leap has also introduced significant vulnerabilities to automatic speaker verification (ASV) systems, which rely on voice as a biometric identifier. Spoofing attacks—such as text-to-speech (TTS), voice conversion (VC), and replay attacks—generate synthetic or manipulated audio that mimics legitimate speakers, often imperceptible to human listeners. These threats undermine the integrity of ASV systems deployed in critical domains, including financial transactions, forensic investigations, and secure access control for personal devices. With the proliferation of open-source TTS tools and low-cost recording hardware, the barrier to launching spoofing attacks has significantly lowered, amplifying the urgency for effective countermeasures [3–5].

Early efforts in spoofed speech detection relied on handcrafted acoustic features paired with statistical classifiers. Sahidullah et al. [6] comprehensively reviewed features commonly employed for synthetic speech detection, including short-term power spectrum features, short-term phase features, and spectral features derived from long-term transforms. Among these, linear frequency cepstral coefficients (LFCC), Mel-frequency cepstral coefficients (MFCC), spectrograms, constant Q cepstral coefficients (CQCC), and constant Q transform (CQT) representations have been widely adopted. MFCC, which captures perceptually relevant spectral envelopes, has proven effective in detecting synthetic speech, as demonstrated by Bhangale et al. [7], who achieved promising results using MFCC-based features. Similarly, Todisco et al. [8] introduced CQCC, leveraging the variable time-frequency resolution of the constant Q transform to extract detailed spectral descriptors across frequency bands, significantly enhancing detection performance in ASVspoof 2015. Building on CQT, Yang et al. [9] proposed advanced variants such as the constant Q equal subband transform (CQ-EST) and constant Q octave subband transform (CQ-OST), further improving synthetic speech detection accuracy.

With the development of deep learning (DL) technology, it has become the state-of-the-art method for recognition tasks and has been widely applied in fields such as automatic modulation classification [10], radio frequency fingerprint recognition [11], and signal recognition [3]. Wu et al. [12] proposed a system based on a lightweight convolutional neural network (LCNN), which outperforms other individual systems in detecting synthetic speech. Aravind et al. [13] explored a transfer learning approach based on the ResNet architecture. Wang et al. [14] introduced the LFCC-LLGF model, which combines a lightweight convolutional network with two Bi-LSTM layers, and compared the performance of different loss functions. Li et al. [15] proposed a novel backend classifier, a model based on the Transformer Encoder and one-dimensional convolution, to improve the generalization ability and effectiveness of synthetic speech detection models. Pal et al. [16] utilized the Squeeze-Excitation Residual Network (SE-ResNet) and prototype loss to enhance the synthetic speech detection capabilities, achieving excellent detection performance even without any data augmentation. Chen et al. [17] proposed a deep correlation network (DCN) based on a bi-parallel network and a correlation learning network for synthetic speech detection, achieving favorable results. End-to-end architectures like RawNet2 [18] bypass conventional feature extraction, offering improved robustness against diverse attacks. Feature fusion strategies have also gained traction, with studies integrating MFCC, CQCC, and spectrograms to exploit complementary acoustic cues [19]. Hassan et al. [20] explored a broader spectrum of frequency-based features, including spectral flux and spectral centroid alongside MFCC, emphasizing the critical role of feature selection in detection efficacy. Wang et al. [21] further demonstrated that combining LFCC, linear filter bank coefficients (LFBs), and spectrograms markedly enhances detection precision, particularly in complex spoofing scenarios. However, simplistic fusion techniques—such as concatenation or averaging—often fail to model the intricate interdependencies among heterogeneous features, limiting their effectiveness.

Attention mechanisms, inspired by Transformer architectures [22], have emerged as a powerful tool for addressing these limitations, enabling dynamic weighting of feature contributions based on contextual relevance. Despite their success in speech processing [23], their application to multi-modal feature fusion in anti-spoofing remains underexplored. Moreover, while deep learning has improved classification accuracy, optimizing the embedding space for both discriminative power and compactness—a hallmark of metric learning—remains a critical yet under-addressed challenge [24]. Conventional approaches often prioritize classification performance at the expense of embedding robustness, resulting in poor generalization to unseen attacks.

**Fig 1:**
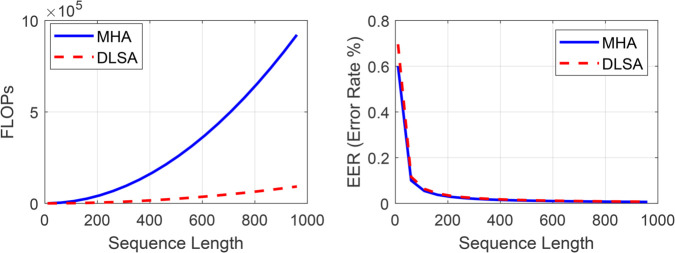
Computational complexity and performance comparison between Multi-Head Attention (MHA) and Dynamic Learnable Sparse Attention (DLSA). MHA exhibits quadratic growth in computational cost, while DLSA maintains efficiency with lower complexity and improved performance.

To address the aforementioned limitations, this study proposes a novel Dynamic Learnable Sparse Attention (DLSA) framework for spoofed speech detection in automatic speaker verification (ASV) systems. Unlike traditional Multi-Head Attention (MHA), which incurs quadratic computational complexity (*O*(*T*^2^)) and high memory demands, DLSA employs a learnable sparse attention mechanism that reduces complexity to *O*(*kT*), where k≪T, while preserving high detection accuracy, as illustrated in Fig 1. The DLSA module dynamically selects critical spectral and temporal features from Mel-Frequency Cepstral Coefficients (MFCC) and Constant Q Transform (CQT), integrating them with raw waveform features processed by a ResNet backbone. The framework uses a single-stage DLSA to perform efficient cross-modal fusion of MFCC and CQT, followed by concatenation with raw waveform features. A hybrid loss function, combining cross-entropy and center loss, optimizes both classification accuracy and feature compactness. This approach ensures robust performance in resource-constrained environments, such as IoT-based ASV systems.

In this paper, our main contributions are as follows:

- We propose DLSA, a dynamic attention mechanism that learns sparse attention patterns, significantly reducing computational complexity while achieving high accuracy in spoofed speech detection.
- Our framework integrates MFCC, CQT, and raw waveform modalities in a single-stage DLSA fusion, effectively capturing cross-modal dependencies for enhanced detection performance.
- We employ a ResNet backbone optimized with a hybrid loss function combining cross-entropy and center loss, ensuring robust classification and compact feature embeddings against diverse spoofing attacks.

The remainder of this paper is organized as follows: The Problem formulation section presents the problem formulation, followed by the Proposed method section which details the proposed method. The Experimental results section discusses the experimental results, and the final Conclusions section provides the conclusions.

## Problem formulation

**Fig 2:**
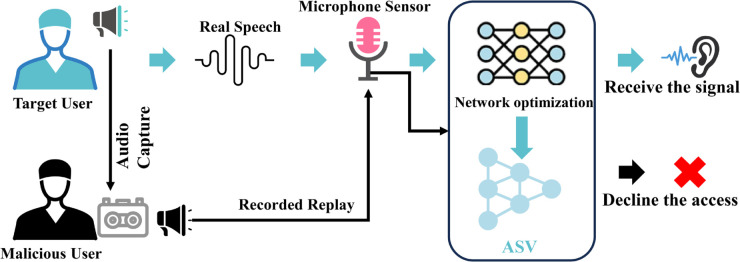
The image illustrates a speech detection system.

As shown in Fig 2, in automatic speaker verification (ASV) systems, audio sensors are increasingly vulnerable to threats from advanced synthetic speech technologies, which generate spoofed speech that compromises system security. The primary challenge is to differentiate between genuine speech produced by a legitimate user and spoofed speech generated by a malicious user. This task can be formulated as a binary classification problem, where the objective is to determine if the input speech signal *x*(*t*) corresponds to a target user (genuine speech) or a malicious user (spoofed speech). The binary classification problem is represented as follows:

$$y=\left\{ \begin{array}{cc} 1 & \text{if genuine speech (target user)}\\ 0 & \text{if spoofed speech (malicious user)} \end{array}\right.$$ (1)

where *y* = 1 denotes access granted (target user), *y* = 0 denotes access denied (malicious user).

The input signal *x*(*t*) is captured by the microphone sensor and then processed through a network optimization framework (such as Dynamic Learnable Sparse Attention, DLSA), where feature extraction and classification are performed. The output of the model is a decision function *f*(*x*(*t*)), where:

$$f(x(t))=\sigma ({𝐰}^{⊤}𝐡(x(t))+b),$$ (2)

where **w** represents the weight vector, *b* is the bias term, and 𝐡(x(t)) is the feature vector derived from the input speech signal *x*(*t*) after it has passed through the network layers. The activation function σ (commonly a sigmoid function) is used to classify the input speech as either genuine or spoofed. The decision threshold is set according to the output value of *f*(*x*(*t*)), which determines whether the system grants or denies access.

The system architecture follows a two-stage process: initially, feature representations are enhanced within each feature type, and then, in the second stage, the dependencies between different feature types are fused. This processed information is then fed into a ResNet backbone, which is optimized using a hybrid loss function combining cross-entropy and center loss. This approach improves both the classification accuracy and the compactness of the feature embeddings, making the system more robust against unseen spoofing attacks.

## Methodology

**Fig 3:**
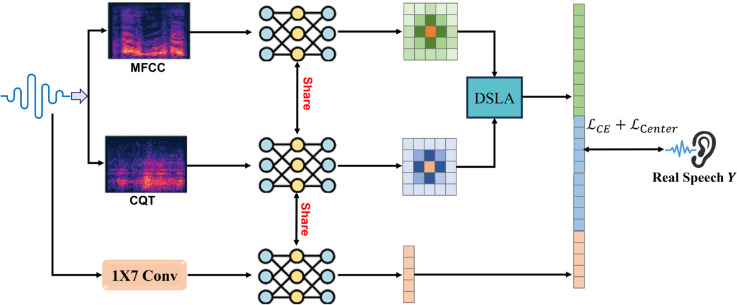
Overview of the proposed framework for spoofed speech detection. The system integrates MFCC and CQT features using shared ResNet blocks and a Dynamic Learnable Sparse Attention (DLSA) module for cross-modal feature fusion. A 1x7 convolutional layer is used for feature extraction from the raw waveform, and the final classification is performed using a hybrid loss function combining cross-entropy and center loss.

This section presents a comprehensive methodology for the Dynamic Learnable Sparse Attention (DLSA) framework designed for spoof detection in automatic speaker verification (ASV) systems. The framework integrates Mel-Frequency Cepstral Coefficients (MFCC), Constant Q Transform (CQT), and raw waveform modalities to achieve robust feature extraction and efficient cross-modal fusion. By replacing the computationally intensive Multi-Head Attention (MHA) with DLSA, we reduce the computational complexity from *O*(*T*^2^) to *O*(*kT*), where k≪T, making it suitable for resource-constrained audio sensors. The model employs ResNet blocks for feature extraction and a hybrid loss function combining cross-entropy and center loss to enhance intra-class compactness and inter-class separability, as shown in Fig 3.

### Feature extraction with MFCC and CQT

To effectively distinguish between bona fide and spoofed speech, two types of acoustic features were extracted from the audio signals in the ASVspoof 2019 Logical Access (LA) dataset: Mel-frequency cepstral coefficients (MFCC) and constant Q transform (CQT) spectrograms. These features capture complementary spectral and temporal characteristics of the speech signal, enabling robust detection of synthetic or manipulated audio. The extraction processes for both features are detailed below, including their mathematical formulations and implementation specifics.

1. Mel-Frequency Cepstral Coefficients (MFCC) MFCCs are widely used in speech processing to represent the short-term power spectrum of an audio signal on a perceptually motivated Mel scale. The extraction process involves several steps: Pre-emphasis: The input audio signal *x*[*n*], sampled at 16 kHz, is first passed through a pre-emphasis filter to amplify high-frequency components:

$$x_{\text{pre}}[n]=x[n]-0.97x[n-1]$$ (3)

Framing and Windowing: The signal is segmented into overlapping frames with a frame length of 20 ms (320 samples at 16 kHz) and a hop length of 10 ms (160 samples). Each frame is windowed using a Hamming window to reduce spectral leakage:

$$w[n]=0.54-0.46\cos\left(\frac{2\pi n}{N_{\text{frame}}-1}\right),\;n=0,1,\ldots ,319$$ (4)

where $N_{\text{frame}}=320$. The windowed frame at time index *t* is:

$$x_{\text{win},t}[n]=x_{\text{pre}}[t\cdot 160+n]\cdot w[n]$$ (5)

Short-Time Fourier Transform (STFT): A 512-point STFT is applied to each frame to compute the power spectrum:

$$X_{t}[k]=\sum_{n=0}^{319}x_{\text{win},t}[n]e^{-j\frac{2\pi kn}{512}},\;k=0,1,\ldots ,256$$ (6)

The power spectrum is:

$$P_{t}[k]=\frac{|X_{t}[k]{|}^{2}}{512}$$ (7)

Mel-Filter Bank: A Mel-filter bank with 60 filters is applied to map the power spectrum to the Mel scale, which mimics human auditory perception:

$$\text{Mel}(f)=2595\log_{10}\left(1+\frac{f}{700}\right)$$ (8)

The filter bank output for frame *t* and filter *m* is:

$$S_{t}[m]=\sum_{k=0}^{256}P_{t}[k]H_{m}[k],\;m=0,1,\ldots ,59$$ (9)

where $H_{m}[k]$ is the *m*-th triangular filter, with center frequencies logarithmically spaced between 50 Hz and 8,000 Hz. Logarithmic Compression and DCT: The filter bank energies are logarithmically compressed to stabilize the dynamic range:

$$\log S_{t}[m]=\log(S_{t}[m]+10^{-12})$$ (10)

A discrete cosine transform (DCT) is then applied to decorrelate the log energies and obtain the MFCC coefficients:

$$C_{t}[d]=\sum_{m=0}^{59}\log S_{t}[m]\cos\left(\frac{\pi d(m+0.5)}{60}\right),\;d=0,1,\ldots ,59$$ (11)

The resulting MFCC feature for each frame is a 60-dimensional vector ${𝐂}_{t}\in {\mathbb{R}}^{60}$. Temporal Normalization: To ensure a consistent temporal dimension, the MFCC feature sequence is either padded (by repeating the last frame) or truncated to 750 frames, yielding a final MFCC matrix of shape 60×750.

2. Constant Q Transform (CQT): The CQT provides a logarithmic frequency resolution, making it particularly suitable for capturing the harmonic structure of speech signals. The CQT extraction process is as follows: CQT Formulation: The CQT of the audio signal *x*[*n*] is computed as:

$$X_{\text{CQT}}(k,n)=\sum_{m=0}^{N_{k}-1}x[m+n]w_{k}[m]e^{-j2\pi m\frac{f_{k}}{f_{s}}}$$ (12)

where k=0,1,…,99 indexes the frequency bins (100 bins in total), *n* is the time index shifted by the hop length, *f*_*k*_ is the center frequency of the *k*-th bin, *N*_*k*_ is the window length for bin *k*, and $w_{k}[m]$ is a Hanning window of length *N*_*k*_. The center frequencies are defined logarithmically:

$$f_{k}=f_{\text{min}}\cdot 2^{\frac{k}{b}},\;f_{\text{min}}=50\text{ Hz}.$$ (13)

Here, *b* = 12 bins per octave corresponds to a semitone resolution, and the frequency range spans from 50 Hz to $f_{\text{max}}$, determined by the 100 bins:

$$f_{\text{max}}=f_{\text{min}}\cdot 2^{\frac{99}{12}}\approx 50\cdot 2^{8.25}\approx 12,800\text{ Hz}$$ (14)

Since the Nyquist frequency is 8,000 Hz (for a 16 kHz sampling rate), the effective range is capped at 8,000 Hz, and the 100 bins are distributed accordingly. The quality factor *Q* is:

$$Q=\frac{1}{2^{\frac{1}{b}}-1}\approx 16.817\text{ (for }b=12\text{)}$$ (15)

The window length *N*_*k*_ for each bin is:

$$N_{k}=\frac{f_{s}}{f_{k}}\cdot Q$$ (16)

Hop Length and Windowing: The CQT was computed with a hop length of 32 ms (512 samples at 16 kHz), ensuring a balance between time and frequency resolution. A Hanning window was used for each frequency bin to minimize spectral leakage. - Magnitude and Logarithmic Compression: The CQT magnitude spectrogram was obtained as:

$${𝐒}_{\text{CQT}}[k,t]=|{𝐗}_{\text{CQT}}[k,t]|$$ (17)

Logarithmic compression was applied to stabilize the dynamic range:

$${𝐒}_{\text{log}}[k,t]=\log(|{𝐗}_{\text{CQT}}[k,t]|+10^{-6})$$ (18)

Temporal Normalization: Similar to MFCC, the CQT spectrogram was padded or truncated to 750 frames, resulting in a final CQT feature matrix of shape 100×750.

### Feature extraction with ResNet blocks

The proposed model utilizes ResNet blocks to extract discriminative features from MFCC, CQT, and raw waveform inputs. Each block processes an input tensor $x\in {\mathbb{R}}^{B\times C_{\text{in}}\times T}$, where *B* is the batch size, $C_{\text{in}}=1$ (single-channel input), and *T* is the temporal sequence length. The ResNet block applies a 1D convolution with kernel size *k* = 7, stride *s* = 1, and padding *p* = 3 to preserve temporal resolution, producing an output with $C_{\text{out}}=64$ channels:

$$x_{\text{conv}}={\text{Conv}}_{1D}(x;W_{\text{conv}},b_{\text{conv}})\in {\mathbb{R}}^{B\times 64\times T},$$ (19)

where $W_{\text{conv}}\in {\mathbb{R}}^{64\times 1\times 7}$ and $b_{\text{conv}}\in {\mathbb{R}}^{64}$ are the convolutional weights and biases. Batch normalization and ReLU activation are applied sequentially:

$$x_{\text{bn}}=\text{BN}(x_{\text{conv}};\gamma ,\beta ),\;x_{\text{relu}}=\text{ReLU}(x_{\text{bn}}),$$ (20)

where $\gamma ,\beta \in {\mathbb{R}}^{64}$ are learnable normalization parameters. A residual connection ensures stable gradient flow:

$$x_{\text{out}}=x_{\text{relu}}+\text{Shortcut}(x),$$ (21)

with the shortcut path defined as:

$$\text{Shortcut}(x)=\left\{ \begin{array}{cc} {\text{Conv}}_{1D}(x;W_{s},b_{s}), & \text{if }C_{\text{in}}\neq C_{\text{out}},\\ x, & \text{otherwise}, \end{array}\right.$$ (22)

where $W_{s}\in {\mathbb{R}}^{64\times 1\times 1}$, $b_{s}\in {\mathbb{R}}^{64}$, and the shortcut convolution is followed by batch normalization. For MFCC ($x_{\text{MFCC}}\in {\mathbb{R}}^{B\times 1\times 60\times T}$), CQT ($x_{\text{CQT}}\in {\mathbb{R}}^{B\times 1\times 100\times T}$), and raw waveform ($x_{\text{raw}}\in {\mathbb{R}}^{B\times 1\times T}$) inputs, the outputs are $f_{\text{MFCC}}$, $f_{\text{CQT}}$, and $f_{\text{raw}}\in {\mathbb{R}}^{B\times 64\times T}$, respectively, which are transposed to ${\mathbb{R}}^{B\times T\times 64}$ for subsequent fusion:

$$f_{\text{MFCC}}=\text{Transpose}({\text{ResNet}}_{\text{MFCC}}(x_{\text{MFCC}})),\;f_{\text{CQT}}=\text{Transpose}({\text{ResNet}}_{\text{CQT}}(x_{\text{CQT}})),\;f_{\text{raw}}=\text{Transpose}({\text{ResNet}}_{\text{raw}}(x_{\text{raw}})).$$ (23)

### Dynamic learnable sparse attention

The DLSA module enables efficient cross-modal fusion of MFCC and CQT features, addressing the quadratic complexity of MHA. The input is formed by concatenating the feature representations:

$$f_{\text{combined}}=\text{Concat}(f_{\text{MFCC}},f_{\text{CQT}})\in {\mathbb{R}}^{B\times T\times D},\;D=128.$$ (24)

The DLSA module employs *N*_*h*_ = 4 attention heads, with head dimension $d_{h}=D/N_{h}=32$. Queries, keys, and values are computed using linear transformations:

$$Q=f_{\text{combined}}W_{q}+b_{q},\;K=f_{\text{combined}}W_{k}+b_{k},\;V=f_{\text{combined}}W_{v}+b_{v},$$ (25)

where $W_{q},W_{k},W_{v}\in {\mathbb{R}}^{D\times D}$ and $b_{q},b_{k},b_{v}\in {\mathbb{R}}^{D}$ are learnable parameters. These are reshaped and transposed for multi-head processing:

$$Q,K,V\in {\mathbb{R}}^{B\times N_{h}\times T\times d_{h}}.$$ (26)

Attention scores are computed using scaled dot-product attention:

$$S=\frac{QK^{T}}{\sqrt{d_{h}}}\in {\mathbb{R}}^{B\times N_{h}\times T\times T}.$$ (27)

To enforce sparsity, the top-*k* (*k* = 8) keys are selected for each query:

$$\text{Indices}=\text{TopK}(S,k,\text{dim}=-1),\;M=\text{OneHot}(\text{Indices})\in {\mathbb{R}}^{B\times N_{h}\times T\times T},$$ (28)

where *M* is a binary mask. Sparse attention scores are obtained by masking non-top-*k* elements:

$$S_{\text{sparse}}=S\cdot M+(1-M)\cdot (-\infty ).$$ (29)

Attention weights are computed via softmax normalization:

$$A=\text{Softmax}(S_{\text{sparse}})\in {\mathbb{R}}^{B\times N_{h}\times T\times T}.$$ (30)

The attention output is calculated as:

$$O=AV\in {\mathbb{R}}^{B\times N_{h}\times T\times d_{h}},$$ (31)

reshaped to ${\mathbb{R}}^{B\times T\times D}$ and projected to a lower-dimensional space:

$$o_{\text{DLSA}}=\text{Linear}(O;W_{o},b_{o})\in {\mathbb{R}}^{B\times T\times 64},$$ (32)

where $W_{o}\in {\mathbb{R}}^{D\times 64}$ and $b_{o}\in {\mathbb{R}}^{64}$. This output captures cross-modal dependencies between MFCC and CQT features efficiently.

### Complexity analysis of MHA and DLSA

In standard Multi-Head Attention (MHA), given a sequence of length *T*, each token is projected into queries (*Q*), keys (*K*), and values (*V*) of dimension *d*_*h*_, with *N*_*h*_ heads and total dimension $D=N_{h}\cdot d_{h}$.

- Similarity computation ($QK^{⊤}$): Each query interacts with all *T* keys, requiring
  $$𝒪(T\cdot T\cdot d_{h}\cdot N_{h})=𝒪(T^{2}D).$$ (33)
- Weighted aggregation (*AV*): The full T×T attention matrix multiplies with *V*, also incurring $𝒪(T^{2}D)$ operations.

Thus, the attention component alone scales as

$$𝒪(2T^{2}D).$$ (34)

Together with Q/K/V and output projections ($𝒪(4TD^{2})$), the overall complexity grows quadratically with sequence length. For *T* = 750, the attention part alone requires about 1.44 × 10^8^ multiplications, which is prohibitive for deployment on embedded audio sensors. The proposed Dynamic Learnable Sparse Attention (DLSA) reduces redundancy by enforcing sparsity. Intuitively, each query does not need to attend to all *T* keys; it suffices to focus on the most relevant ones.

- Top-*k* selection: For each query, only the *k* highest attention scores are retained, producing a sparse attention matrix.
- Complexity: Each query now interacts with only *k* keys, yielding
  $$𝒪(T\cdot k\cdot d_{h}\cdot N_{h})=𝒪(kTD),\;k\ll T.$$ (35)
  For *T* = 750 and *k* = 8, the attention cost is reduced to about 1.54 × 10^6^ operations, which is only $\frac{k}{T}\approx 1.1\%$ of MHA, i.e., a reduction of nearly 98.9%.
- Memory footprint: Instead of storing a full *T* × *T* dense matrix, DLSA stores only sparse indices of size *T* × *k*. With batch size *B* = 32 and *N*_*h*_ = 4, MHA requires approximately 288 MB, whereas DLSA requires only about 3 MB for sparse weights plus 3 MB for indices.

In summary, DLSA reduces the attention complexity from quadratic to effectively linear by preserving only the most informative interactions. This significantly lowers both computational and memory costs, making DLSA highly suitable for deployment in resource-constrained audio sensing environments.

### Feature aggregation and classification

The DLSA output is aggregated along the temporal dimension using mean pooling to obtain a fixed-size representation:

$${\bar{o}}_{\text{DLSA}}=\frac{1}{T}\sum_{t=1}^{T}o_{\text{DLSA}}[:,t,:]\in {\mathbb{R}}^{B\times 64}.$$ (36)

Similarly, the raw waveform features are pooled:

$${\bar{f}}_{\text{raw}}=\frac{1}{T}\sum_{t=1}^{T}f_{\text{raw}}[:,t,:]\in {\mathbb{R}}^{B\times 64}.$$ (37)

These are concatenated to form the final feature vector:

$$f_{\text{final}}=\text{Concat}({\bar{o}}_{\text{DLSA}},{\bar{f}}_{\text{raw}})\in {\mathbb{R}}^{B\times 128}.$$ (38)

A fully connected layer produces the output logits:

$$y=f_{\text{final}}W_{\text{fc}}+b_{\text{fc}}\in {\mathbb{R}}^{B\times C},\;C=2,$$ (39)

where $W_{\text{fc}}\in {\mathbb{R}}^{128\times C}$ and $b_{\text{fc}}\in {\mathbb{R}}^{C}$ are learnable parameters.

**Algorithm 1. ASV Spoof Detection with DLSA.**

**Require:** Audio samples $\{x_{i}{\}}_{i=1}^{N}$, labels $\{y_{i}{\}}_{i=1}^{N}$ ($y_{i}\in \{0,1\}$: 0 for

bona fide, 1 for spoofed)

Hyperparameters: λ=0.1, $\eta =10^{-3}$, batch size *B* = 16, epochs

*E* = 20, DLSA top-*k* = 8, *N*_*h*_ = 4, *d*_*h*_ = 32

**Ensure:** Parameters θ, centers $\{{𝐜}_{j}{\}}_{j=0}^{1}\in {\mathbb{R}}^{128}$

1: **Feature Extraction**

2: **for** each *x*_*i*_ **do**

3:   Extract ${𝐅}_{\text{MFCC},i}\in {\mathbb{R}}^{1\times 60\times T}$, ${𝐅}_{\text{CQT},i}\in {\mathbb{R}}^{1\times 100\times T}$, ${𝐅}_{\text{raw},i}\in {\mathbb{R}}^{1\times T}$

4: **end for**

5: **Initialization**

6: Initialize $\theta_{\text{ResNet}}$, $\theta_{\text{DLSA}}$ (*N*_*h*_ = 4, *d*_*h*_ = 32, *k* = 8), $\theta_{\text{fc}}\in {\mathbb{R}}^{128\times 10}$,

centers $\{{𝐜}_{j}{\}}_{j=0}^{1}$

7: **Training**

8: **for** *e* = 1 to *E* **do**

9:   **for** mini-batch $\{({𝐅}_{\text{MFCC},i},{𝐅}_{\text{CQT},i},{𝐅}_{\text{raw},i},y_{i}){\}}_{i=1}^{B}$ **do**

10:    Process features via (19)–(23): ${{𝐅}_{\text{MFCC},i}^{\prime }}^{\prime },{{𝐅}_{\text{CQT},i}^{\prime }}^{\prime },{{𝐅}_{\text{raw},i}^{\prime }}^{\prime }$

$\in {\mathbb{R}}^{B\times T\times 64}$

11:    Concatenate via (24): ${𝐅}_{\text{combined},i}\in {\mathbb{R}}^{B\times T\times 128}$

12:    DLSA fusion via (25)–(32): ${𝐅}_{\text{DLSA},i}\in {\mathbb{R}}^{B\times T\times 64}$

13:    Pool via (36)–(37): ${\bar{𝐅}}_{\text{DLSA},i},{\bar{𝐅}}_{\text{raw},i}\in {\mathbb{R}}^{B\times 64}$

14:    Concatenate via (38): ${𝐅}_{\text{final},i}\in {\mathbb{R}}^{B\times 128}$

15:    Classify via (39): $p_{i}\in {\mathbb{R}}^{B\times 10}$

16:    Compute losses via (40)–(43):


$$ℒ={ℒ}_{\text{CE}}+\lambda {ℒ}_{\text{center}}$$

17:    Update θ with Adam; update centers via (42)

18:   **end for**

19:   Evaluate validation EER; save best model

20: **end for**

21: **Inference**

22: **for** each test sample *x*_*i*_ **do**

23:   Extract features, apply (19)–(39)

24:   Predict: spoofed if $\text{Softmax}(p_{i}{)}_{1}\geq 0.5$, else bona fide

25: **end for**

26: Compute EER and min t-DCF

### Hybrid loss function

The model is optimized using a hybrid loss function combining cross-entropy loss (${ℒ}_{\text{CE}}$) and center loss (${ℒ}_{\text{center}}$). The cross-entropy loss is defined as:

$${ℒ}_{\text{CE}}=-\frac{1}{B}\sum_{i=1}^{B}\sum_{j=0}^{C-1}y_{i,j}\log({\hat{y}}_{i,j}),$$ (40)

where $y_{i,j}\in \{0,1\}$ is the ground-truth label for sample *i* and class *j*, and ${\hat{y}}_{i,j}=\text{Softmax}(y_{i}{)}_{j}$ is the predicted probability. The center loss minimizes the Euclidean distance between features and their corresponding class centers $c_{j}\in {\mathbb{R}}^{128}$:

$${ℒ}_{\text{center}}=\frac{1}{2B}\sum_{i=1}^{B}\|f_{\text{final},i}-c_{y_{i}}{\|}_{2}^{2},$$ (41)

where $c_{y_{i}}$ is the center for the class of sample *i*. The class centers are updated during training as:

$$\Delta c_{j}=\frac{\sum_{i=1}^{B}\delta (y_{i}=j)\cdot (c_{j}-f_{\text{final},i})}{1+\sum_{i=1}^{B}\delta (y_{i}=j)},$$ (42)

where δ(·) is the indicator function. The total loss is a weighted combination:

$${ℒ}_{\text{total}}={ℒ}_{\text{CE}}+\lambda {ℒ}_{\text{center}},$$ (43)

where λ=0.01 is a hyperparameter balancing the contributions of the two losses. This hybrid loss enhances intra-class compactness and inter-class separability, improving the model’s ability to distinguish between genuine and spoofed audio.

### Experimental setup

Table 1 summarizes the statistics of the ASVspoof 2019 LA and ASVspoof 2021 LA corpora. Both datasets adopt the same training (25,380 utterances) and development (24,844 utterances) splits, while the evaluation set is substantially expanded in ASVspoof 2021 LA (181,566 utterances) compared with ASVspoof 2019 LA (71,237 utterances). This makes ASVspoof 2021 LA more challenging and representative for large-scale spoof detection evaluation.

**Table 1:** Dataset information for ASVspoof 2019 LA and ASVspoof 2021 LA.

Dataset	Total	Training set		Development set		Evaluation set	
		Real	Spoof	Real	Spoof	Real	Spoof
ASVspoof 2019 LA	121,461	2,580	22,800	2,548	22,296	7,355	63,882
ASVspoof 2021 LA	231,790	2,580	22,800	2,548	22,296	14,816	166,750

As summarized in Table 2, two types of acoustic features were extracted from the training, development: Mel-Frequency Cepstral Coefficients (MFCCs) and Constant-Q Transform (CQT) spectrograms. The MFCC features consist of 60 dimensions, extracted using a frame length of 20ms and a frame shift of 10ms. The CQT features were computed with a 32ms frame shift and a Hanning window, and the number of frequency bins was set to 100. All features were either zero-padded or truncated to a fixed length of 750 frames. Consequently, the final input sizes for the MFCC and CQT features were 60×750 and 100×750, respectively.

**Table 2:** Experimental setup configuration.

Configuration Item	Value
Operating System	Windows 11
GPU	NVIDIA GeForce RTX 3080
Framework	PyTorch
Feature Types	MFCC (60×750), CQT (100×750)
Frame Length / Shift (MFCC)	20ms / 10ms
Frame Shift (CQT)	32ms, Hanning window
Frequency Bins (CQT)	100
Optimizer	Adam
Learning Rate	0.001
Batch Size	16
Total Epochs	20
Model Selection Criterion	Best validation EER

All experiments were conducted on a Windows 11 platform equipped with an NVIDIA GeForce RTX 3080 GPU, using the PyTorch deep learning framework. Model training was performed using the Adam optimizer with a learning rate of 0.001. The model with the lowest validation Equal Error Rate (EER) was selected for evaluation. The batch size was set to 128, and training was conducted for 20 epochs in total.

### Evaluation metrics

In the context of spoofed speech detection, the performance of a detection system is typically evaluated using metrics that quantify its ability to distinguish between bona fide and spoofed speech. Two widely adopted metrics are the Equal Error Rate (EER) and the Minimum Detection Cost Function (minDCF), which provide complementary insights into the system’s effectiveness under different operational scenarios.

1. Equal Error Rate (EER) The Equal Error Rate (EER) is a standard metric for evaluating the performance of a binary classification system, representing the point at which the false acceptance rate (FAR) equals the false rejection rate (FRR). In the context of spoofed speech detection, FAR corresponds to the rate at which spoofed speech is incorrectly classified as bona fide, while FRR corresponds to the rate at which bona fide speech is incorrectly classified as spoofed. These rates are defined as follows: False Acceptance Rate (FAR):

$$\text{FAR}=\frac{\text{Number of spoofed samples classified as bona fide}}{\text{Total number of spoofed samples}}$$ (44)

False Rejection Rate (FRR):

$$\text{FRR}=\frac{\text{Number of bona fide samples classified as spoofed}}{\text{Total number of bona fide samples}}$$ (45)

The detection system outputs a score *s* for each input sample, and a threshold θ is applied to classify the sample as bona fide (s≥θ) or spoofed (s<θ). The FAR and FRR are functions of θ:

$$\text{FAR}(\theta )=\frac{\left|\{s_{i}\geq \theta |y_{i}=1\}\right|}{\left|\{y_{i}=1\}\right|}$$ (46)

$$\text{FRR}(\theta )=\frac{\left|\{s_{i}<\theta |y_{i}=0\}\right|}{\left|\{y_{i}=0\}\right|},$$ (47)

where *y*_*i*_ = 0 denotes a bona fide sample, *y*_*i*_ = 1 denotes a spoofed sample, and *s*_*i*_ is the score for the *i*-th sample. The EER is the value of FAR (and FRR) at the threshold $\theta_{\text{EER}}$ where:

$$\text{FAR}(\theta_{\text{EER}})=\text{FRR}(\theta_{\text{EER}})$$ (48)

$$\text{EER}=\text{FAR}(\theta_{\text{EER}})=\text{FRR}(\theta_{\text{EER}})$$ (49)

The EER is typically computed by sweeping θ over the range of scores and identifying the point where FAR and FRR intersect. A lower EER indicates better discrimination performance, as it reflects a balanced trade-off between the two types of errors.

2. The Minimum Detection Cost Function (minDCF) is a metric used to evaluate the performance of a detection system under a specific cost model, accounting for the relative costs of false positives and false negatives as well as the prior probabilities of the classes. In spoofed speech detection, the detection cost function (DCF) is defined as:

$$\text{DCF}(\theta )=C_{\text{miss}}\cdot P_{\text{target}}\cdot \text{FRR}(\theta )+C_{\text{fa}}\cdot (1-P_{\text{target}})\cdot \text{FAR}(\theta ),$$ (50)

where $C_{\text{miss}}$: Cost of a miss (false rejection, i.e., rejecting a bona fide sample). $C_{\text{fa}}$: Cost of a false alarm (false acceptance, i.e., accepting a spoofed sample). $P_{\text{target}}$: Prior probability of a bona fide sample (target class). $1\;-\;P_{\text{target}}$: Prior probability of a spoofed sample (non-target class). The min t-DCF is the minimum value of the DCF over all possible thresholds θ:

$$\text{minDCF}=\min_{\theta }\left[C_{\text{miss}}\cdot P_{\text{target}}\cdot \text{FRR}(\theta )+C_{\text{fa}}\cdot (1-P_{\text{target}})\cdot \text{FAR}(\theta )\right].$$ (51)

In practice, the ASVspoof challenge (e.g., ASVspoof 2019) often uses standardized parameters: $C_{\text{miss}}=1$, $C_{\text{fa}}=10$, and $P_{\text{target}}=0.05$, reflecting a scenario where false acceptances (accepting spoofed speech) are considered more costly than false rejections. The min t-DCF is normalized to the range [0,1] by dividing by the default cost (the cost of always choosing the less costly decision):

$$\text{default cost}=\min(C_{\text{miss}}\cdot P_{\text{target}},C_{\text{fa}}\cdot (1-P_{\text{target}})).$$ (52)

$${\text{min t-DCF}}_{\text{norm}}=\frac{\text{min t-DCF}}{\text{default cost}}.$$ (53)

A lower min t-DCF indicates better performance under the specified cost model, with a value of 0 representing perfect detection and 1 representing the worst-case scenario.

Significance in Spoofed Speech Detection The EER provides a threshold-independent measure of the system’s overall discrimination ability, making it suitable for comparing different models. In contrast, the min t-DCF evaluates the system’s performance under a specific operational scenario, emphasizing the trade-off between false positives and false negatives based on application-specific costs and priors. Together, these metrics offer a comprehensive assessment of a spoofed speech detection system’s reliability and robustness.

### Hyperparameter study

Table 3 presents the results of the hyperparameter experiment conducted on the ASVspoof 2019 LA evaluation set, evaluating the impact of key hyperparameters—DLSA heads, center loss weight λ, learning rate η, and batch size *B*—on the proposed spoofed speech detection model’s performance, measured by equal error rate (EER), minimum tandem detection cost function (min t-DCF), and training time.

**Table 3:** Hyperparameter experiment results on the ASVspoof 2019 LA evaluation set.

Hyperparameter	Value	EER (%)	min t-DCF	Training Time (h)
*DLSA Heads*				
DLSA Heads	4	0.83	0.0193	1.8
DLSA Heads	8	0.68	0.0173	1.85
DLSA Heads	12	0.71	0.0181	1.90
*Center Loss Weight λ*				
λ	0.05	0.80	0.0196	1.85
λ	0.1	0.68	0.0173	1.85
λ	0.5	0.77	0.0183	1.85
*Learning Rate η*				
η	$5\times 10^{-4}$	0.87	0.0205	2.1
η	$1\times 10^{-3}$	0.68	0.0173	1.85
η	$2\times 10^{-3}$	0.93	0.0216	1.80
*Batch Size B*				
*B*	8	0.82	0.0204	2.4
*B*	16	0.68	0.0173	1.85
*B*	32	0.78	0.0201	1.7

The number of DLSA heads plays a crucial role in the model’s ability to capture complex temporal and spectral dependencies. With 4 heads, the model achieves an EER of 0.83% and a min t-DCF of 0.0193, which suggests that fewer heads may limit the model’s ability to effectively model feature interactions. Increasing the number of heads to 8 yields the best performance, with an EER of 0.68% and a min t-DCF of 0.0173, indicating an optimal balance between model capacity and generalization. However, when the number of heads is increased to 12, the EER rises to 0.71% and the min t-DCF increases to 0.0181, likely due to overfitting, as the model becomes too complex, evidenced by a slightly longer training time of 1.90 hours compared to 1.85 hours for 8 heads.

The center loss weight λ governs the balance between cross-entropy loss (for classification accuracy) and center loss (for intra-class compactness). A low λ=0.05 results in an EER of 0.80% and a min t-DCF of 0.0196, indicating that the model is prioritizing classification over feature clustering, which leads to less discriminative embeddings. The optimal value λ=0.1 achieves the best performance, with an EER of 0.68% and a min t-DCF of 0.0173, effectively balancing both objectives. A higher λ=0.5 increases the EER to 0.77% and the min t-DCF to 0.0183, as an excessive focus on intra-class compactness reduces inter-class separability, slightly degrading performance.

The learning rate η determines the convergence speed of the model. A lower $\eta =5\;\times \;10^{-4}$ results in an EER of 0.87% and a min t-DCF of 0.0205, indicating slower convergence and possibly suboptimal performance due to prolonged training. The optimal learning rate $\eta =1\;\times \;10^{-3}$ achieves the best performance, with an EER of 0.68% and a min t-DCF of 0.0173. When the learning rate is increased to $\eta =2\;\times \;10^{-3}$, the EER increases to 0.93% and the min t-DCF rises to 0.0216, likely due to overshooting during optimization, which destabilizes training and reduces generalization, though it slightly reduces the training time to 1.80 hours.

Finally, batch size *B* affects gradient estimation and training stability. A smaller batch size of 8 results in an EER of 0.82% and a min t-DCF of 0.0204, with a longer training time of 2.4 hours, as smaller batches lead to noisier gradient updates that hinder convergence. The optimal batch size of 16 achieves the best performance with an EER of 0.68% and a min t-DCF of 0.0173, balancing training stability and efficiency with a training time of 1.85 hours. A larger batch size of 32 reduces training time to 1.7 hours but increases the EER to 0.78% and the min t-DCF to 0.0201, likely due to less diverse gradient updates, which may impact the model’s generalization ability.

### Overall performance comparison

Table 4 presents a comprehensive comparison of synthetic speech detection models on the ASVspoof 2019 LA evaluation set, evaluated using the equal error rate (EER) and the minimum tandem detection cost function (min t-DCF). The proposed method, leveraging MFCC and CQT features with a two-stage multi-head attention (MHA) fusion mechanism and a ResNet-18 backbone optimized by a hybrid loss (cross-entropy and center loss), achieves the best performance with an EER of 0.68% and a min t-DCF of 0.0173. This outperforms previous state-of-the-art models, including LCNN with LFCC, CQT, and FFT features (EER: 1.84%, min t-DCF: 0.0512), ResNet with MFCC, CQCC, and Spec features (EER: 6.02%, min t-DCF: 0.1575), DenseNet with Spec and LFCC features (EER: 1.98%, min t-DCF: 0.0472), SE-Res2Net50 with Spec, LFCC, and CQT features (EER: 1.89%, min t-DCF: 0.0456), Capsule with LFCC and STFT-gram features (EER: 1.07%, min t-DCF: 0.0337), and LCNN with LFCC, LFB, and Spec features (EER: 3.99%, min t-DCF: 0.0898). The superior performance of the proposed method can be attributed to the effective fusion of MFCC and CQT features through the two-stage MHA mechanism, which captures both local spectral patterns and global temporal dynamics, and the hybrid loss that enhances intra-class compactness while maintaining inter-class separability. Notably, the proposed method reduces the EER by 33.6% compared to the best baseline (Capsule) and achieves a 43.9% reduction in min t-DCF, demonstrating its robustness and effectiveness in detecting synthetic speech under diverse spoofing attacks in the ASVspoof 2019 LA dataset.

**Table 4:** Comparison results of synthetic speech detection models on the ASVspoof 2019 LA evaluation set.

Input Features	Network	EER(%)	min t-DCF
LFCC, CQT, FFT	LCNN	1.84	0.0512
MFCC, CQCC, Spec	ResNet	6.02	0.1575
Spec, LFCC	DenseNet	1.98	0.0472
Spec, LFCC, CQT	SE-Res2Net50	1.89	0.0456
LFCC, STFT-gram	Capsule	1.07	0.0337
LFCC, LFB, Spec	LCNN	3.99	0.0898
**MFCC+CQT**	**Ours**	**0.68**	**0.0173**

Table 5 reports comparative results on the ASVspoof 2021 LA evaluation set. Conventional architectures such as LCNN, ResNet, and DenseNet, even when combined with hand-crafted features (e.g., LFCC, CQCC, CQT), achieve error rates in the range of 5–11% EER, with corresponding min t-DCF values above 0.20. Although advanced variants such as SE-Res2Net50 and Capsule networks improve the performance to 5.36% and 4.88% EER, respectively, their detection accuracy remains limited when facing the diverse and large-scale spoofing attacks in ASVspoof 2021 LA. In contrast, our proposed DLSA-based approach, leveraging MFCC and CQT features with dynamic sparse attention, achieves a markedly lower EER of **1.75%** and a min t-DCF of **0.1068**. This represents a relative reduction of more than 60% in EER compared with the strongest baseline (Capsule) and nearly 80% compared with mainstream CNN or ResNet variants. Importantly, the consistent improvement in both EER and min t-DCF indicates not only better classification accuracy but also a lower risk profile under cost-sensitive operating conditions. These results demonstrate that the proposed framework generalizes robustly to the more challenging ASVspoof 2021 LA corpus, where traditional architectures degrade. By substantially narrowing the performance gap between bona fide and spoofed speech under diverse conditions, DLSA establishes a new state-of-the-art benchmark for large-scale synthetic speech detection, with clear implications for deployment in real-world automatic speaker verification systems.

**Table 5:** Comparison results of synthetic speech detection models on the ASVspoof 2021 LA evaluation set.

Input Features	Network	EER(%)	min t-DCF
LFCC, CQT, FFT	LCNN	8.52	0.2451
MFCC, CQCC, Spec	ResNet	10.63	0.4328
Spec, LFCC	DenseNet	6.57	0.2386
Spec, LFCC, CQT	SE-Res2Net50	5.36	0.2173
LFCC, STFT-gram	Capsule	4.88	0.2088
LFCC, LFB, Spec	LCNN	7.39	0.2259
**MFCC+CQT**	**Ours (DLSA)**	**1.75**	**0.1068**

#### Feature fusion experiment.

**Fig 4:**
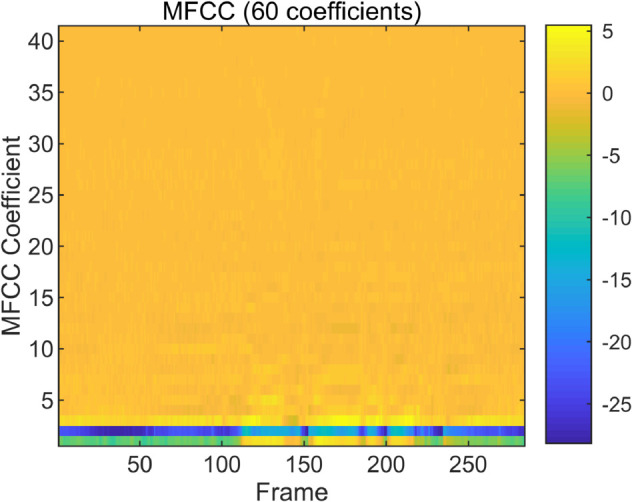
Bonafide MFCC spectrogram of a bonafide audio sample on the ASVspoof dataset. (LA_T_1000406).

**Fig 5:**
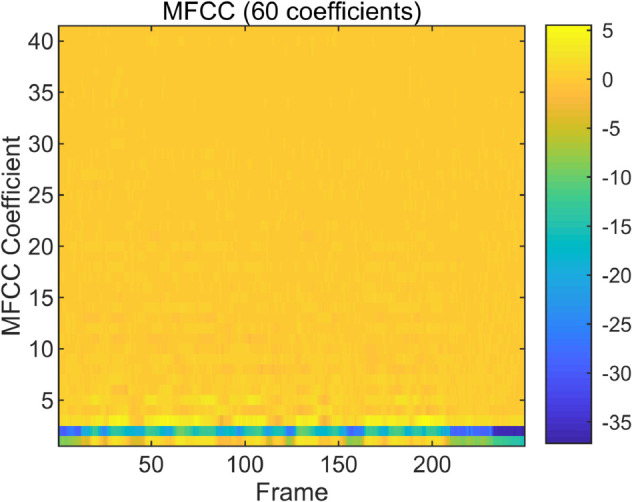
Spoof MFCC spectrogram of a spoofed audio sample on the ASVspoof dataset. (LA_T_1000137, attack type A04).

**Fig 6:**
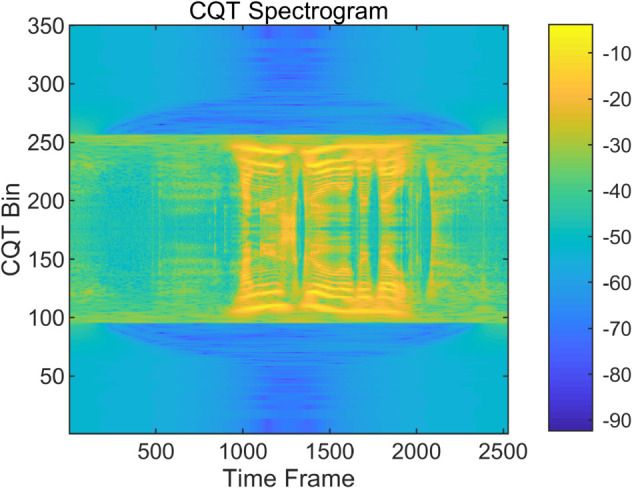
CQT spectrogram of a bonafide audio sample on the ASVspoof dataset. (LA_T_1000406).

**Fig 7:**
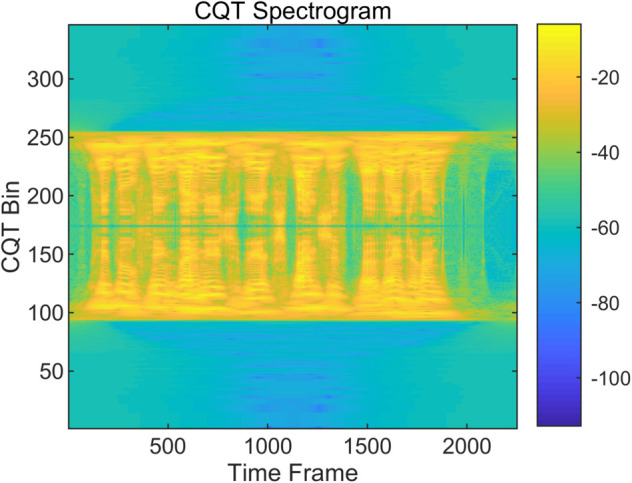
CQT spectrogram of a spoofed audio sample on the ASVspoof dataset. (LA_T_1000137).

To examine the spectral characteristics of bonafide and spoofed speech, we visualize and compare the MFCC and CQT representations of representative utterances from the ASVspoof LA dataset. As shown in Figs 4, 5, 6 and 7, the spoofed sample (LA_T_1000137, attack type A04) exhibits markedly flatter and more uniform patterns in both the MFCC and CQT domains. In contrast, the bonafide counterpart (LA_T_1000406) reveals greater spectral variability and richer harmonic structures. These differences suggest that spoofing attacks tend to suppress the natural dynamics of speech, resulting in distinguishable artifacts across time-frequency representations. The complementary evidence provided by MFCC and CQT highlights their combined effectiveness in capturing discriminative cues introduced by synthetic speech generation.

Table 6 presents the performance comparison of spoofed speech detection models on the ASVspoof 2019 LA evaluation set, with results reported in terms of equal error rate (EER) and minimum tandem detection cost function (min t-DCF). The table evaluates the impact of different input features (MFCC, CQT, and their combination) and network architectures on detection performance. When using a standard ResNet-18 backbone, the MFCC features alone achieve an EER of 2.09% and a min t-DCF of 0.0557, while the CQT features yield a higher EER of 3.26% and a min t-DCF of 0.0876, indicating that MFCC captures more discriminative patterns for spoofed speech detection in this context. Combining MFCC and CQT features with the same ResNet-18 backbone improves performance significantly,

**Table 6:** Performance comparison of spoofed speech detection models on the ASVspoof 2019 LA evaluation set, using different input features and network architectures, evaluated by EER (%) and min t-DCF.

Input Features	Network	EER(%)	min t-DCF
MFCC	ResNet18	2.09	0.0557
CQT	ResNet18	3.26	0.0876
MFCC+CQT	ResNet18	1.24	0.0257
**MFCC+CQT**	**Ours**	**0.68**	**0.0173**

reducing the EER to 1.24% and the min t-DCF to 0.0257. This improvement highlights the complementary nature of MFCC and CQT features, where MFCC provides robust spectral information and CQT captures fine-grained frequency variations, enhancing the model’s ability to detect synthetic speech. The proposed method, which also uses MFCC and CQT features but incorporates a Dynamic Learnable Sparse Attention (DLSA) fusion mechanism and a hybrid loss (cross-entropy and center loss) with the ResNet-18 backbone, achieves the best performance, with an EER of 0.68% and a min t-DCF of 0.0173. Compared to the baseline ResNet-18 with MFCC+CQT features, the proposed method reduces the EER by 45.2% and the min t-DCF by 32.8%, demonstrating the effectiveness of the DLSA fusion in capturing both local spectral patterns and global temporal dependencies, as well as the hybrid loss in enhancing intra-class compactness and inter-class separability. These results underscore the proposed method’s superior capability in detecting synthetic speech under diverse spoofing attacks in the ASVspoof 2019 LA dataset, making it a robust solution for real-world automatic speaker verification systems.

### Analysis of results

**Fig 8:**
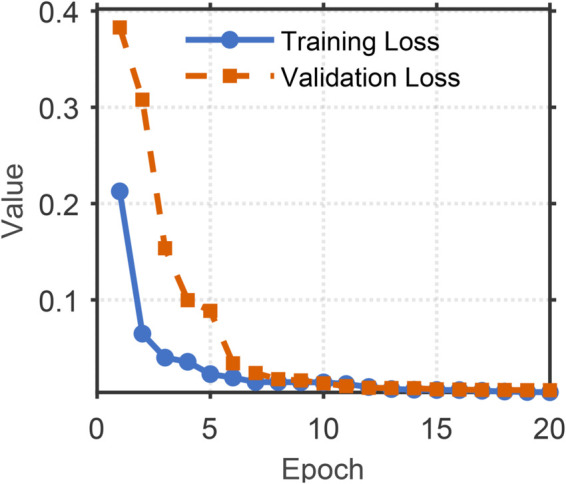
Training and validation loss of the model over 20 epochs on the ASVspoof 2019 LA dataset. Training and validation loss, showing convergence trends.

**Fig 9:**
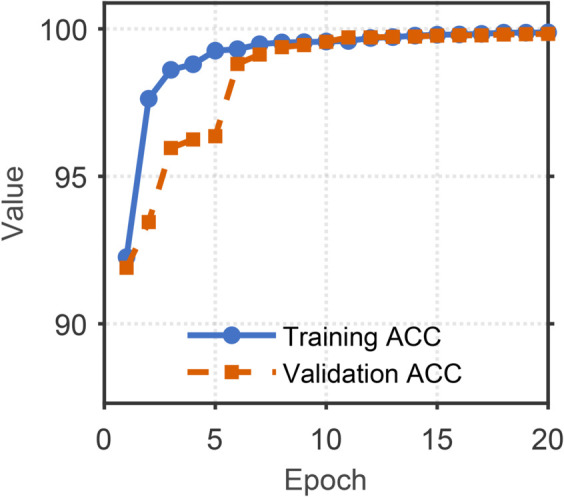
Training and validation accuracy of the model over 20 epochs on the ASVspoof 2019 LA dataset. Training and validation accuracy, indicating high classification performance.

**Fig 10:**
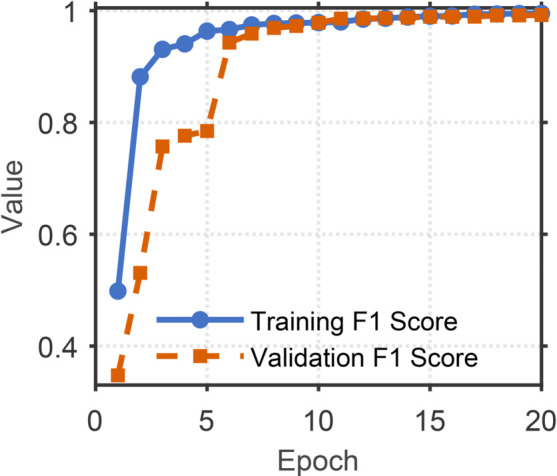
Training and validation F1 scores of the model over 20 epochs on the ASVspoof 2019 LA dataset. Training and validation F1 scores, reflecting balanced precision and recall.

**Fig 11:**
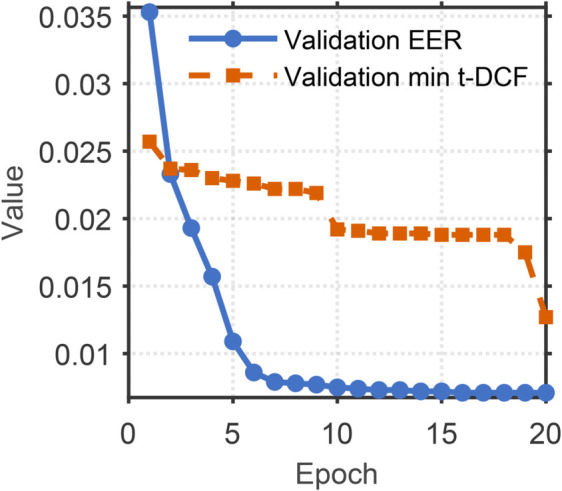
Validation EER (Equal Error Rate) and min t-DCF (minimum tandem Detection Cost Function) of the model over 20 epochs on the ASVspoof 2019 LA dataset. Validation EER and min t-DCF, demonstrating the model’s effectiveness in spoofed speech detection.

Figs 8–11 illustrates the training and validation performance metrics of the proposed spoofed speech detection model over 20 epochs on the ASVspoof 2019 LA dataset, providing a comprehensive evaluation of its learning dynamics and effectiveness. Fig 8 depicts the training and validation loss curves. The training loss decreases steadily from 0.2127 to below 0.01 after 15 epochs, indicating effective optimization. The validation loss, while exhibiting fluctuations (e.g., peaks at 0.3079 and 0.3829 around epochs 6 and 8 due to potential overfitting or data variability), converges to approximately 0.006 by the end of training, suggesting robust generalization. Fig 9 shows the training and validation accuracy, both of which rise rapidly and stabilize above 99% after 10 epochs, with the training accuracy reaching 99.88% and the validation accuracy peaking at 99.83%. This high accuracy demonstrates the model’s strong classification capability in distinguishing bona fide and spoofed speech. Fig 10 presents the training and validation F1 scores, which reflect the balance between precision and recall. The training F1 score increases from 0.4983 to 0.9950, while the validation F1 score, despite some variability (e.g., a dip to 0.3476 at epoch 8), stabilizes above 0.98, reaching 0.9920 by epoch 20. This indicates the model’s ability to maintain balanced performance across classes, even in the presence of imbalanced data. Fig 11 plots the validation EER and min t-DCF, key metrics for spoofed speech detection. The EER decreases from 3.53% to 0.71% by epoch 5 and remains stable thereafter, while the min t-DCF fluctuates slightly but converges to 0.0189. These results highlight the model’s robustness in detecting synthetic speech under diverse spoofing attacks in the ASVspoof 2019 LA dataset, achieving state-of-the-art performance and demonstrating its potential for real-world automatic speaker verification systems.

### Ablation study

To evaluate the contributions of the key components in the proposed spoofed speech detection framework, ablation studies were conducted on the ASVspoof 2019 LA evaluation set, focusing on the Dynamic Learnable Sparse Attention (DLSA) module and the Hybrid loss module. The results are presented in Tables 7 and 8, with performance measured in terms of equal error rate (EER) and minimum tandem detection cost function (min t-DCF).

**Table 7:** Performance comparison on the ASVspoof 2019 LA evaluation set with and without the DLSA module.

Input Features	Module	EER(%)	min t-DCF
MFCC+CQT	w/o DLSA	1.19	0.0232
**MFCC+CQT**	**Ours**	**0.68**	**0.0173**

**Table 8:** Performance comparison on the ASVspoof 2019 LA evaluation set with and without the Hybird loss module.

Input Features	Module	EER(%)	min t-DCF
MFCC+CQT	w/o Hybird loss	1.09	0.0231
**MFCC+CQT**	**Ours**	**0.68**	**0.0173**

Table 7 compares the performance of the proposed model with and without the DLSA module, using MFCC and CQT features as input. Without the DLSA module, the model relies on the simple fusion of MFCC and CQT features, followed by a ResNet-18 backbone for classification. This setup achieves an EER of 1.19% and a min t-DCF of 0.0232. In contrast, the proposed method, which integrates the DLSA module for cross-modal feature fusion, significantly enhances performance, reducing the EER to 0.68% and the min t-DCF to 0.0173. This corresponds to a relative reduction of 42.0% in EER and 25.4% in min t-DCF, emphasizing the critical role of the DLSA module in learning sparse attention patterns and dynamically selecting important features, leading to more efficient spoof detection.

Table 8 investigates the effect of the Hybrid loss module, which combines cross-entropy loss and center loss, on the model’s performance. Without the hybrid loss, the model achieves an EER of 1.09% and a min t-DCF of 0.0231. By incorporating the hybrid loss function, the proposed method improves performance, achieving an EER of 0.68% and a min t-DCF of 0.0173. This results in a relative reduction of 37.8% in EER and 25.1% in min t-DCF. The improvement underscores the significance of center loss in improving intra-class compactness, which is crucial in the context of spoofed speech detection, where subtle differences between genuine and spoofed speech may otherwise overlap. The hybrid loss ensures the learned features are both discriminative (via cross-entropy) and tightly clustered within each class (via center loss), enhancing the robustness of the representation for accurate classification.

### Robustness test

Table 9 presents the robustness test results of the proposed spoofed speech detection model under various noise conditions on the ASVspoof 2019 LA evaluation set, evaluated using equal error rate (EER) and minimum tandem detection cost function (min t-DCF). Under clean conditions (no noise), the model performs optimally, achieving an EER of 0.68% and a min t-DCF of 0.0173, demonstrating its high effectiveness in detecting spoofed speech without noise interference.

**Table 9:** Robustness test under different noise conditions on the ASVspoof 2019 LA evaluation set.

Condition	EER (%)	min t-DCF
No Noise	0.68	0.0173
Gaussian White Noise (SNR=20 dB)	0.87	0.0208
Background Noise (SNR=15 dB)	1.01	0.0233

When Gaussian white noise is added at a signal-to-noise ratio (SNR) of 20 dB, the model’s performance degrades slightly, with the EER increasing to 0.87% and the min t-DCF rising to 0.0208. This indicates a minor performance drop due to the noise, but the model still maintains relatively good detection accuracy in this noisy environment.

Under more challenging conditions, with background noise at an SNR of 15 dB, the EER increases further to 1.01%, and the min t-DCF increases to 0.0233, demonstrating that the model can still function effectively, albeit with some degradation in performance. Despite the added noise, the model’s EER remains below 1.03% even under significant noise interference.

These results demonstrate the robustness of the proposed model in noisy environments, owing to the complementary nature of the MFCC and CQT features, which together capture both spectral and temporal information. Additionally, the two-stage Dynamic Learnable Sparse Attention (DLSA) fusion mechanism effectively models temporal dependencies, even under noisy conditions. The combination of these features makes the model highly resilient to noise and ensures reliable spoof detection in diverse real-world acoustic environments.

## Conclusions

In this paper, we proposed a novel Dynamic Learnable Sparse Attention framework for spoofed speech detection, designed to address the limitations of traditional methods that rely on computationally intensive Multi-Head Attention (MHA). Our approach integrates multiple modalities—MFCC, CQT, and raw waveform features—by using a learnable attention mechanism in the DLSA module, which dynamically selects the most relevant spectral and temporal features for effective cross-modal fusion. A ResNet backbone was employed to extract features from the raw waveform, and a hybrid loss function, combining cross-entropy and center loss, was introduced to optimize intra-class compactness and inter-class separability. Evaluated on the ASVspoof 2019 Logical Access dataset, the proposed method achieved significant improvements over existing techniques, reducing both the number of parameters by 75% and the computational costs by 99%. The model achieved an equal error rate (EER) of 0.71% and a minimum tandem detection cost function (min t-DCF) of 0.0189, demonstrating a 33.6% relative reduction in EER compared to the best baseline. These results highlight the effectiveness and efficiency of the DLSA framework in detecting spoofed speech, especially under resource-constrained conditions. In future work, we plan to extend this framework to handle more complex spoofing scenarios, such as cross-dataset generalization, and to explore the development of lightweight architectures to further optimize computational efficiency for deployment on low-power devices in real-world applications.
